# PyOncoPrint: a python package for plotting OncoPrints

**DOI:** 10.5808/gi.22079

**Published:** 2023-03-31

**Authors:** Jeongbin Park, Nagarajan Paramasivam

**Affiliations:** 1 School of Biomedical Convergence Engineering, Pusan National University, Busan 50612, Korea; 2 Computational Oncology, Molecular Precision Oncology Program, National Center for Tumor Diseases (NCT), German Cancer Research Center (DKFZ), Heidelberg 69120, Germany

**Keywords:** OncoPrint, plotting, Python

## Abstract

OncoPrint, the plot to visualize an overview of genetic variants in sequencing data, has been widely used in the field of cancer genomics. However, still, there have been no Python libraries capable to generate OncoPrint yet, a big hassle to plot OncoPrints within Python-based genetic variants analysis pipelines. This paper introduces a new Python package PyOncoPrint, which can be easily used to plot OncoPrints in Python. The package is based on the existing widely used scientific plotting library Matplotlib, the resulting plots are easy to be adjusted for various needs.

## Introduction

OncoPrint, the plot to visualize an overview of the genetic variants of the deposited data at cBioPortal [1,2], has become popular, especially in the field of cancer genomics [3-7]. Although OncoPrints can be easily drawn and exported at the cBioPortal website, it is difficult to generate OncoPrints via the website from the command-line-based bioinformatics workflows. To tackle this problem, there has been an R implementation [8] to plot OncoPrints, however, still there have been no Python implementations so far. We introduce a novel Python package, PyOncoPrint, for plotting OncoPrints in Python. PyOncoPrint supports various scenarios of plotting OncoPrints, such as plotting metadata, variant statistics, etc. alongside the main OncoPrint, so that it can be directly used as a figure of a paper with no modifications.

## Methods

### Implementation

The package is mainly based on Matplotlib [9], the de facto standard Python plotting library. The variant markers are plotted using the ‘scatter’ function of Matplotlib, which enables the plotting of all scatter plot marker shapes available in the Matplotlib package. In addition to conventional markers, custom markers can be designed by defining polygon coordinates. Thus, one can define as many marker shapes for printing various types of genetic variants.

**Fig. 1.**
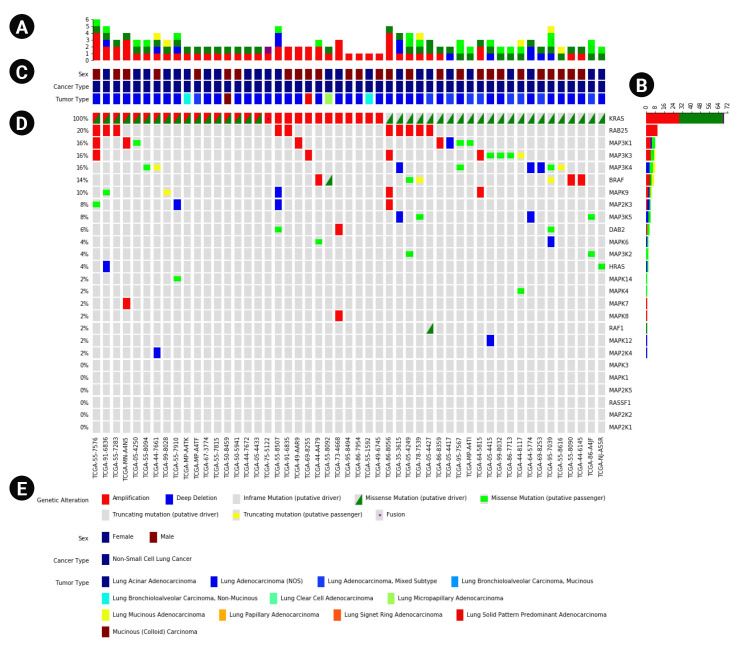
An example OncoPrint based on The Cancer Genome Atlas data downloaded from cBioPortal. (A, B) The top and right plots that show the frequency of variants per type. (C) The annotations panel that displays metadata of samples. (D) The main OncoPrint. (E) The plot legend including the variant markers and annotations.

In addition to the main plot (Fig. 1D), PyOncoPrint supports the plotting of subplots to provide more information. One of them is the ‘annotations’ plot (Fig. 1C) attached to the top of the main Oncoprint, which plots sample metadata. The annotations include any categorical information of samples, such as sex, tumor type, etc. The annotations can be printed as a legend, and attached to the bottom of the plot (Fig. 1E). The other subplots are ‘top plot’ and ‘right plot,’ bar plots that summarize the frequency of the variants of samples (Fig. 1A) and genes (Fig. 1B).

PyOncoPrint can automatically sort the samples and genes by the frequency of each genetic variant so that one can easily overview the plotted genetic variants, and be ready to use as a figure in a research paper. The genetic variation data can be easily imported as Pandas data frame [10], as well as the CSV files exported from cBioPortal can be directly used as an input.

### Basic usage

The input data to the PyOncoPrint is Pandas dataframe which contains a matrix of samples vs. genes. The input follows the format of cBioPortal’s CSV exports—each element of the matrix defines variants as strings, concatenated with commas. Thus, one can either generate their own data or just convert a cBioPortal’s export to a Pandas dataframe, as an input to PyOncoPrint. By providing the input data with marker definitions and annotations, just one simple function ‘pyoncoprint’ generates the OncoPrint. A detailed example, that shows the basic usage of PyOncoPrint—including how to define the input data, markers, and annotations—is available online on our GitHub repository.

## Results

We demonstrated visualization of OncoPrint of The Cancer Genome Atlas lung adenocarcinoma data using PyOncoPrint. The data was obtained from the Oncoprinter at cBioPortal, containing 24 genes and 996 patient samples as a tab-delimited format.

The downloaded data was then loaded as a Pandas dataframe object using the “read_csv” function of Pandas. The patients having no mutations in the 24 genes were truncated, resulting in 463 remaining patients having at least one mutation.

Next, the marker types for each mutation pattern were defined. For demonstration purpose, three different marker types for the mutation types were defined as following: (1) fill patterns, which fills the marker with a specified color and given height; (2) an asterisk symbol (*); and (3) a custom triangle pattern defined using “Polygon” class available in Matplotlib. All of the markers were defined with different colors so that the mutation types could be distinguishable from each other.

Finally, the plot was generated using “oncoprint” method of PyOncoPrint (Supplementary Fig. 1), which shows the mutational landscape of the lung adenocarcinoma patients.

## Conclusion

We developed a novel Python package, PyOncoPrint, which provides an easy way to plot OncoPrints using Python. Thanks to its simple usage and easy-to-use interface, the package can be easily adapted to various Python-based command-line pipelines. The source code is freely available on our GitHub repository (https://github.com/pnucolab/pyoncoprint).

## Supplementary Materials

Supplementary Figure 1. The OncoPrint of The Cancer Genome Atlas lung adenocarcinoma data, generated by PyOncoPrint. The figure shows 24 genes and 463 patients having 11 different mutilation types, including amplification (red, fill), deep deletion (blue, fill), putative driver splice mutation (dark orange, fill with half height), putative passenger splice mutation (orange, fill with half height), putative driver structural variant (purple, asterisk), putative passenger structural variant (fuchsia, asterisk), putative driver inframe mutation (brown, fill with half height), putative driver missense mutation (green, triangle), putative passenger missense mutation (lime, fill with half height), putative driver truncating mutation (black, fill with half height), and putative passenger truncating mutation (yellow, fill with half height).
