# Supplementary material for: PyOncoPrint: a python package for plotting OncoPrints
Source: Genomics Inform. 2023 Mar 31;21(1):e14. doi: 10.5808/gi.22079 (PMC10085746; doi:10.5808/gi.22079)
Supplement: Supplementary Figure 1. — The OncoPrint of The Cancer Genome Atlas lung adenocarcinoma data, generated by PyOncoPrint. The figure shows 24 genes and 463 patients having 11 different mutilation types, including amplification (red, fill), deep deletion (blue, fill), putative driver splice mutation (dark orange, fill with half height), putative passenger splice mutation (orange, fill with half height), putative driver structural variant (purple, asterisk), putative passenger structural variant (fuchsia, asterisk), putative driver inframe mutation (brown, fill with half height), putative driver missense mutation (green, triangle), putative passenger missense mutation (lime, fill with half height), putative driver truncating mutation (black, fill with half height), and putative passenger truncating mutation (yellow, fill with half height). [file gi-22079-Supplementary-Figure-1.pdf]

TCGA Lung Adenocarcinoma (PanCancer Atlas), default sort method

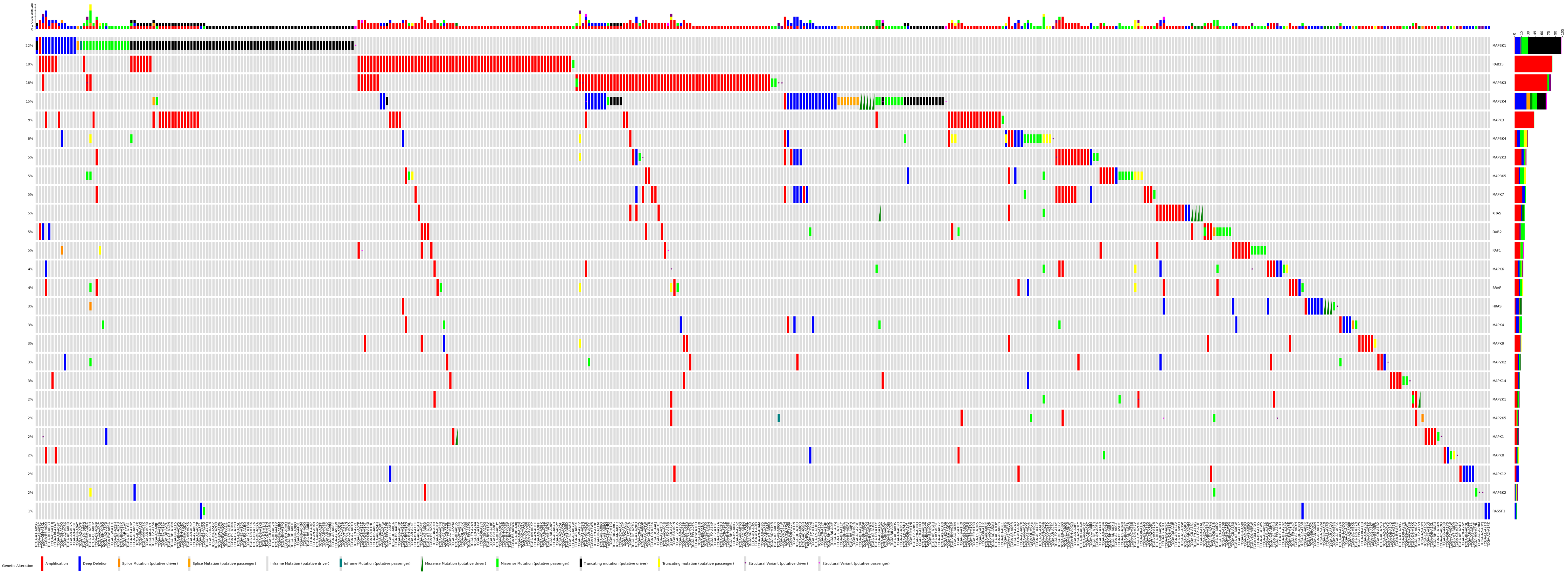

**Supplementary Fig. 1.** The OncoPrint of The Cancer Genome Atlas lung adenocarcinoma data, generated by PyOncoPrint. The figure shows 24 genes and 463 patients having 11 different mutilation types, including amplification (red, fill), deep deletion (blue, fill), putative driver splice mutation (dark orange, fill with half height), putative passenger splice mutation (orange, fill with half height), putative driver structural variant (purple, asterisk), putative passenger structural variant (fuchsia, asterisk), putative driver inframe mutation (brown, fill with half height), putative driver missense mutation (green, triangle), putative passenger missense mutation (lime, fill with half height), putative driver truncating mutation (black, fill with half height), and putative passenger truncating mutation (yellow, fill with half height).
